# A Natural History Study of *RP2*-Related Retinopathy

**DOI:** 10.3390/jcm11236877

**Published:** 2022-11-22

**Authors:** Riccardo Cheloni, Daniel Jackson, Mariya Moosajee

**Affiliations:** 1 UCL Institute of Ophthalmology, London EC1V 9EL, UK; 2 Moorfields Eye Hospital NHS Foundation Trust, London EC1V 2PD, UK; 3 The Francis Crick Institute, London NW1 1AT, UK; 4 Great Ormond Street Hospital for Children NHS Foundation Trust, London WC1N 3JH, UK

**Keywords:** retinitis pigmentosa, X-linked retinitis pigmentosa, *RP2*, inherited retinal diseases, natural history, genetic eye disease, optical coherence tomography, OCT

## Abstract

X-linked retinitis pigmentosa (RP) is a severe form of RP, often with early macular involvement. This study aimed to characterise the natural history of patients with a diagnosis of X-linked RP due to *RP2* mutations. Clinical details, best-corrected visual acuity (BCVA) and multimodal retinal imaging were retrospectively collected from patients with *RP2* variants from Moorfields Eye Hospital (London, UK). Measures of the ellipsoid-zone (EZ) width, central retinal thickness (CRT), and thickness of the photoreceptor and retinal pigment epithelium complex (PR+RPE, taken between the external limiting membrane and RPE) were extracted from spectral-domain optical coherence tomography (SD-OCT) scans. A total of 47 affected males (median baseline age: 20 years, IQR: 12.5–36.5) were included, and 41 had two or more visits (median follow-up: 8.0 years, IQR: 3.2–14.5). A total of 24 *RP2* variants were identified, 13 of which were novel. BCVA dropped from 0.66 LogMAR at baseline (IQR, 0.35–1.4) to 1.3 LogMAR at the most recent visit (IQR: 0.6–1.4). SD-OCT revealed a prevalent outer retinal atrophy (n = 23/35, 65.7%), and measurable EZ width at baseline in 34.3% of patients (n = 12). Age significantly affected all quantitative measures (*p* < 0.001) except EZ width (*p* = 0.58), with exponential decays of 46–49% and 12.6–33.9% per decade for BCVA and SD-OCT measures, respectively. *RP2* patients exhibited rapid progression to outer retina atrophy and early macular involvement with substantial vision loss by age 30–40.

## 1. Introduction

Retinitis pigmentosa (RP) is a heterogeneous group of inherited retinal diseases, characterised by a progressive degeneration of rods, followed by cones and retinal pigment epithelium (RPE) [1]. RP is the most common retinal dystrophy with a prevalence of 1 in 4000 [2] and represents a major cause of visual impairment in the working-age population [3,4,5]. Inheritance can be autosomal dominant, autosomal recessive or X-linked, with the latter representing up to 20% of all forms of RP [6,7]. Up to five genes causing X-linked RP have been identified [8,9], with *RPGR* (retinitis pigmentosa GTPase regulator) and *RP2* (retinitis pigmentosa 2) accounting for the majority of cases [10,11]. *RP2* was the second gene discovered to be causing X-linked RP [12], and its variants are responsible for 10–20% of X-linked RP [12,13]. *RP2* (OMIM #312600) consists of 5 exons, 1050 base pairs and encodes a protein of 350 amino acid residues, acting as a GTPase-activating protein for Arf-like protein 3. It is ubiquitously expressed in plasma membranes throughout the retina, including photoreceptors and RPE, and has a central role in cellular transport regulation. Up to 133 disease-associated variants of *RP2* have been discovered, yet the molecular mechanism resulting in degeneration of the human retina is not fully understood [9,14], and current animal models have generally shown milder phenotypes compared to humans [15].

X-linked RP has an earlier onset and more severe phenotype than autosomal cases of RP with nyctalopia in early childhood and progressive visual field constriction, then extensive central vision loss and blindness by age 40 [16,17]. Many clinical measures appear similar between *RP2* and *RPGR*-related retinopathy [9], yet a few reports may suggest more severe involvement of central vision in *RP2* [16,17]. Previous work has shown widespread signs of macular involvement, with 36% of patients presenting with a central scotoma before the age of 12 [17].

Gene therapy is now a realistic option for patients with molecularly confirmed biallelic *RPE65*-retinopathy, which aims to restore protein expression via adeno-associated viral (AAV) vector delivery of healthy *RPE65* cDNA. There is no gene-directed therapy for *RP2*, but X-linked RPs are a suitable target for developing gene therapies, being monogenic diseases [18,19]. The interest in X-linked RPs has been further enhanced by encouraging findings in animal and cellular models showing some level of preserved function and promising rescue effects [20,21,22,23]. For example, an AAV carrying a photoreceptor-specific human *RP2* gene expression cassette produced stable *RP2* protein expression in a knockout mice model, with preservation of cone function over 18 months duration [22].

Natural history studies are important for guiding the planning of clinical trials, informing the selection of participants and the choice of outcome measures capable of depicting disease progression in a timely and accurate manner. Accordingly, there are many studies assessing the natural history of *RPGR* [18,24,25], but there is a paucity of information regarding *RP2* patients, with most studies conducted on small samples and/or with cross-sectional designs [14,16,17,26]. In this study, we aimed to characterise the natural history of patients with a molecular diagnosis of X-linked *RP2*-retinopathy in a quantitative longitudinal analysis focused on retinal structure. The results provide insights into the development of outcome measures for future clinical trials, the selection of suitable candidates for receiving treatment and better support for patient counselling on prognosis.

## 2. Material and Methods

### 2.1. Settings and Study Population

This was a retrospective longitudinal case series at a single tertiary referral centre (Moorfields Eye Hospital NHS Foundation Trust, London, UK). Potential subjects were identified from the prospectively consented Moorfields Eye Hospital Inherited Eye Disease Database for structure/function of genetic diseases (Research Ethics Number: 12/LO/0141) and all procedures adhered to the tenets of the Declaration of Helsinki. Data for these studies are collected as part of standard of care and retrospectively analysed.

Patients with molecular genetic confirmation of disease-causing variants of *RP2* were identified. The pathway of genetic testing and variant interpretation at Moorfields has been previously described extensively [6,27,28]. Referred patients presenting clinical findings suggestive of genetic eye diseases are offered genetic testing of either single gene, targeted gene panels, or whole genome sequencing. The results of genetic testing undergo thorough bioinformatic processing and quality checks and then each identified variant is analysed to establish its possible association with the disease phenotype. Several steps are taken here to ensure the quality and reliability of genetic testing before variants are filtered using a minor allele frequency <0.001 in publicly available and in-house data sets, predicted protein effect, and familial segregation. Genetic results are then reviewed by a multidisciplinary panel including clinical geneticists, molecular biologists and clinicians managing the family to confirm variant pathogenicity [29] and to establish a molecular diagnosis.

For all subjects, a full ophthalmic assessment was conducted at each visit as part of their clinical care, including best-corrected visual acuity (BCVA), refraction where appropriate and retinal imaging. Data were extracted from the electronic medical records of each patient and supplemented with written records, where appropriate. Visual field and electrophysiology examinations were available in a minority of patients, mainly at a single time point and showing a severe loss at baseline. Accordingly, these were not considered in this analysis and BCVA was the sole functional parameter assessed.

### 2.2. Retinal Imaging

Spectral-domain optical coherence tomography (SD-OCT), (19 B-scans, 512 A-scans/B-scans; 97 B-scans, 1024 A-scans/B-scans) and fundus autofluorescence (FAF) imaging were performed using a Heidelberg Spectralis (Heidelberg Engineering, Heidelberg, Germany) with Automated Retinal Tracking. The central OCT B-scan was identified by a trained observer as having the least residual inner retinal tissue and the thickest outer nuclear layer presence. FAF imaging was performed using high-power blue light autofluorescence at 30° or 55° depending on which best visualised the residual FAF area. Wide-field pseudo-colour fundus photography was performed with the Optos California (Optos plc, Dunfermline, UK).

Consideration of OCT exams was limited to gradable scans, which were deemed to be so if enabling assessment of fovea centration (i.e., multiple B-scans centred in the macula) and not substantially affected by artefacts due to floaters, poor optical quality, ocular movements or blinks. The classification proposed by Bouzia et al. [30] was used to qualitatively assess ellipsoid zone (EZ) layers in gradable scans, including: (1) continuous and intact EZ, (2) focally disrupted EZ, (3) focally disrupted EZ with RPE changes, and (4) severely disrupted EZ with RPE changes or thinning. The presence of complications, such as epiretinal membranes and cystoid macular oedema (CMO), was also recorded. EZ width and retinal thickness were measured using the inbuilt measuring tool within the Heidelberg Spectralis image viewer by a senior trained grader (Figure S1). Retinal thickness was measured manually as the vertical distance between retinal layers of interest at the fovea for consistency across scans and to avoid a mismatch between axial and lateral resolution [31]. Central retinal thickness (CRT) was measured between the inner limiting membrane and the posterior border of the RPE. As previously proposed, photoreceptor and RPE complex (PR+RPE) thickness was recorded as a measure of outer retinal thickness [32] and taken between the external limiting membrane and RPE. EZ width was measured manually as the sum of nasal and temporal EZ widths, identified at the anterior border of the EZ layer.

Fundus photos were graded qualitatively for the presence of key clinical findings of RP [1]. FAF was assessed for patterns consistent with hyper-autofluorescent ring, central hyper-autofluorescence or severely decreased autofluorescence [33].

### 2.3. Data Analysis

VAs were converted to LogMAR, and measures of counting fingers, hand movement, light perception and no light perception were converted to 2.6, 2.7, 2.8 and 2.9 LogMAR, respectively [32,34]. VA scores of counting fingers or worse were excluded from further progression rate analysis as this would represent end-stage disease with limited utility as an outcome metric in a trial setting [35]. The inter-ocular relationship was assessed with Spearman’s correlation between quantitative clinical measures. Bias and 95% limits of agreement according to Bland–Altman were also computed [36]. Given the high degree of human inter-ocular symmetry, measurements from the right eye were used for further descriptive and quantitative analyses.

Longitudinal data were not available for all patients, hence, we adopted two approaches to assess changes in quantitative measures over time. Simple linear regression was used to assess age-related changes from cross-sectional examinations at baseline. In a longitudinal approach we considered only data from patients with two visits or more and used mixed models to account for repeated measures [37]. We built isolated models for each quantitative measure, passing the clinical parameter of interest (e.g., BCVA) as the outcome measure, age at visit as a fixed effect and patient ID as a random effect (intercept). As suggested before [38], we also modelled age-related loss with exponential decay by computing the natural logarithm of the outcome variable of interest. For this analysis, BCVAs data were converted to letter scores and effect sizes were converted back to LogMAR.

We conducted survival analysis for quantitative outcome measures with Kaplan–Meier curves [39]. An outcome of legal blindness was considered for BCVA (≥1.0 LogMAR, corresponding to the threshold for sight impairment registration in the UK), and a finding of non-measurable width was considered for EZ width. For CRT and PR+RPE thickness we considered the upper limit of RPE normative thickness (mean ± standard deviation: 17.6 µm ± 1.9 µm) [40], and endpoints were achieved if CRT and PR+RPE were smaller than 22 µm (1.96 SD above mean). For each outcome, we applied left and right censoring, and age at the time of the visit was passed as a time variable.

All statistical analyses were performed in R [41] and statistical significance was considered if *p* < 0.05. Where appropriate, family-wise Bonferroni correction was applied to control for multiple testing.

## 3. Results

We included 47 male affected patients from 33 unrelated families with X-linked RP due to mutations of *RP2*. Details of demographic and clinical characteristics are reported in Table 1. Data on ethnicity were not available for the majority of patients. Most data series did not follow a normal distribution when represented visually from histograms and assessed by Shapiro–Wilk tests, and therefore median and interquartile range (IQR) were adopted as summary statistics.

### 3.1. Molecular Characteristics

Across all 47 patients, 24 variants were identified in total (Figure S2 and Table S1). Thirteen variants were novel with five nonsense — c.19A>T p.(Lys7*), c.181C>T p.(Gln61*), c.258T>A p.(Cys86*), c.450G>A p.(Trp150*) and c.460G>T p.(Glu154*); six frameshift—c.128_140del13 p.(Ser43Metfs*3), c.128delG p.(Ser43Metfs*3), c.159_160insAA p.(Pro54Asnfs*5), c.235delG p.(Ala79Leufs*11), c.568_569delinsG p.(Pro190Glufs*48) and c.685_691del7 (p.Gln229Alafs*7); 1 splicesite (c.969+3A>T) and 1 missense c.341G>A p.(Cys114Tyr)). Variants c.352C>T p.(Arg118Cys) and c.358C>T p.(Arg120*) were the most frequent, affecting five patients in two unrelated families and five patients in five unrelated families, respectively.

### 3.2. Visual Acuity

**Figure 1:**
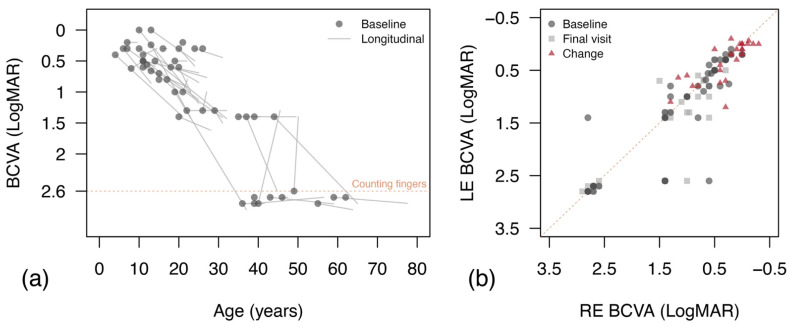
Changes in best corrected visual acuity (BCVA) by age and inter-ocular relationship. (**a**) BCVA measures at the first visit from all patients are reported as data points. For patients with follow-up visits, the best linear fit for all visits was reported for each patient (light grey segments). The orange line reports the BCVA level for counting fingers; (**b**) reports the inter-ocular relationship between the BCVAs of the right (RE) and left eyes (LE) at baseline, at last visit and the intervening change.

Measures of BCVA at baseline were available for all patients (n = 47, Table 1), being counting fingers or worse in 10 patients. There were 41 patients with two or more visits, who could be considered for longitudinal analysis (median number of visits: 2, IQR: 2 to 5, max: 14), with a median follow-up time of 8.0 years (IQR: 3.2 to 14.5). Median BCVA at baseline was 0.66 LogMAR (IQR 0.35 to 1.4), dropping to a median of 1.3 LogMAR (IQR: 0.6 to 1.4) at the last visit. Inter-ocular relationship analysis (Figure 1b) showed a strong correlation between BCVA scores in right and left eyes at baseline (Spearman’s rho: 0.90, 95%CI: 0.80 to 0.95, *p* < 0.0001) and last visit (Spearman’s rho: 0.81, 95%CI: 0.63 to 0.91, *p* < 0.0001). BCVA changes from baseline were also strongly related (Spearman’s rho: 0.78, 95%CI: 0.56 to 0.89, *p* < 0.0001). Findings from Bland–Altman analysis of agreement are reported in Table S2. No bias was observed for BCVA measures, and 95% limits of agreements ranged between 0.8 and 1.1 LogMAR.

As reported in Figure 1a, there were significant changes in BCVA with age, and cross-sectional analysis from baseline examinations showed a linear effect of age of β: 0.33 LogMar/10 years (95%CI: 0.22 to 0.44, *p* < 0.0001, R^2^: 0.523). Modelling data with exponential decay provided a better fit, with a rate of loss of −46.0% per 10 years (95%CI: −58.3 to −33.5, *p* < 0.0001, R^2^: 0.606). A slightly stronger impact of age on BCVA was observed in the longitudinal analysis, with a linear effect of age on BCVA of β: 0.37 LogMAR/10 years (95%CI: 0.32 to 0.43, *p* < 0.0001, R^2^: 0.544). Similar fitting was achieved with exponential decay, with a rate loss of −49.0% per 10 years (95%CI: −56.2 to −41.8, *p* < 0.0001, R^2^: 0.554).

### 3.3. Ocular Imaging

Pseudo-colour fundus photos were available for 40 patients (85.1%, median age 25 years, IQR: 19.75 to 37) and gradable for 37 patients. Details of the grading are reported in Table 1. Pigment deposition in the mid or far periphery (bone-spicule or pigment clumping) and attenuated blood vessels were prevalent findings in this cohort, being present in 89.2% and 94.6% of patients, respectively. Notably, macular abnormalities were detectable in the fundus photos of 22 patients (58.5%), and those presenting visible modifications were on average older (32 years, IQR: 19.3 to 39.8 vs 11 years IQR: 8.5 to 14, Mann–Whitney test, *p* = 0.004), and had poorer BCVA (1.4 LogMAR, IQR: 0.61 to 2.7, vs 0.3 LogMAR, IQR: 0.3 to 0.45, Mann–Whitney test, *p* = 0.0005) compared to patients without macular changes.

**Figure 2:**
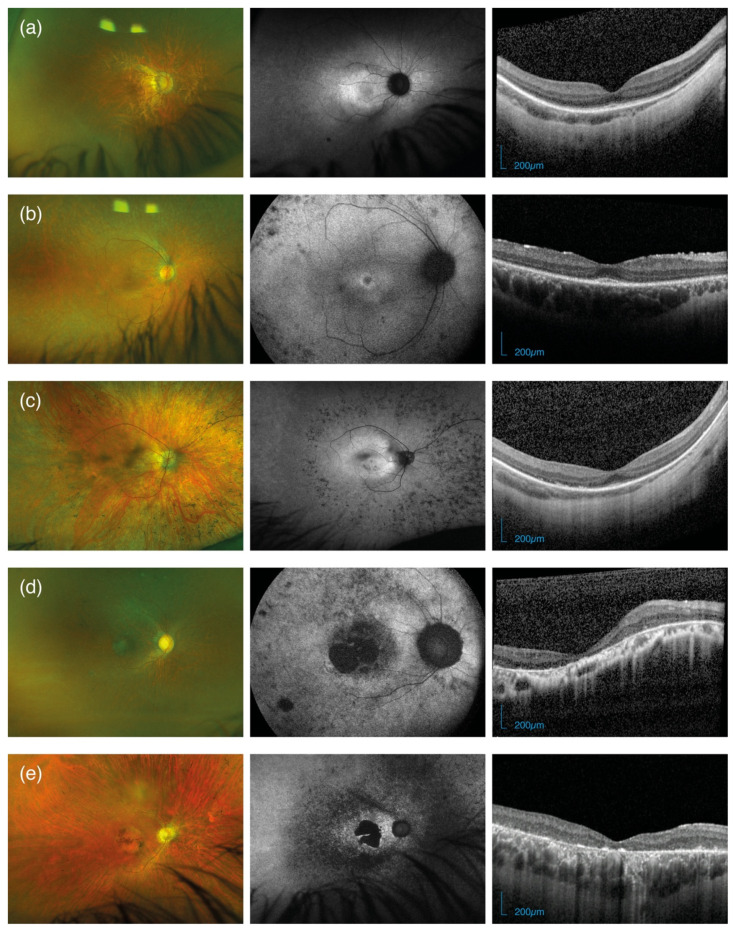
Optos pseudo-colour fundus photos, fundus autofluorescences and SD-OCT scans for five male patients with *RP2*-retinopathy with increasing age and severity: (**a**) 12-year-old patient (ID: 15222–3, Table S1) with c.341G>A mutation; (**b**) 15-year-old patient (ID: 20948–3) with c.352C>T mutation; (**c**) 25-year-old patient (ID: 20016–2) with c.14_16delTCT mutation; (**d**) advanced case of a 27-year-old patient with c.685_691del7 mutation (ID: 5284–2) and (**e**) a 31-year-old with c.568_569delinsG mutation (ID: 49–2).

FAF was obtained for 43 patients (91.5%, median age: 23.5 years, IQR: 19 to 33.5), being gradable for 38 of these. Details of the grading are summarised in Table 1, and a pattern consistent with a hyper auto-fluorescent ring was recognised in nine patients (23.8%). These patients were of younger age compared to those with central hyper autofluorescence or severe loss (15 years, IQR: 12 to 16, vs 26 years, IQR: 20 to 43, Mann–Whitney test, *p* = 0.0004) and with better BCVA (0.4 LogMAR, IQR: 0.3 to 0.62, vs 1.0 LogMAR, IQR: 0.5 to 1.4, Mann–Whitney test, *p* = 0.03). Seven of the nine patients (77.8%) with a FAF ring pattern also had measurable EZ width in SD-OCT scans, with a median width of 633 µm (IQR: 472.5 to 910.5). The majority of patients (n = 21, 55.3%), however, had severe loss of autofluorescence, whereas two patients (5.3%) showed granular mottled patches of hypo-autofluorescence limited to the periphery, with seemingly intact FAF at the posterior pole. Examples of multimodal imaging for five representative patients with *RP2*-related retinal dystrophy are reported in Figure 2.

### 3.4. SD-OCT Quantitative Measures

At least one SD-OCT exam was available for all 42 patients (median age: 23 years IQR: 18 to 35.8), being gradable in 35 of these (median age: 21 years IQR: 17.5 to 27). Among the non-gradable scans, six patients were imaged with a single line scan pattern (1 B-scan) whereby the foveal position could not be ascertained. For the remaining patients, poor scan quality prevented the detection of the fovea and assessment of centration. There were 27 patients with two or more follow-up visits and usable SD-OCTs for longitudinal analysis, overall resulting in a median of 4 visits (IQR: 3 to 6) and a median follow-up time of 6 years (IQR: 3 to 7). For baseline visits in right eyes, one patient presented CMO (2.8%, age 17 years), whereas epiretinal membranes were a common finding, being detected in 17 patients (48.5%, median age 24 years IQR: 19.5 to 28).

#### 3.4.1. Ellipsoid Zone

**Figure 3:**
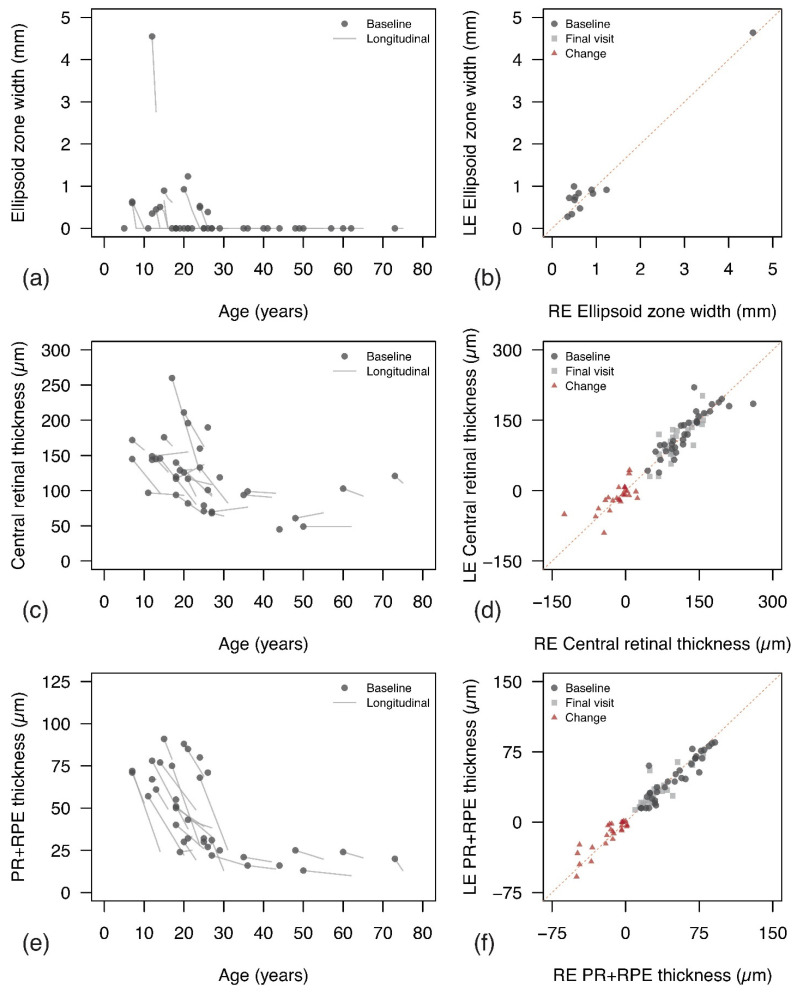
Changes in SD-OCT quantitative measures by age (**a**,**c**,**e**) and inter-ocular relationship (**b**,**d**,**f**) for ellipsoid zone (EZ) width, central retinal thickness (CRT), and photoreceptor and retinal pigment epithelium complex (PR+RPE) thickness. The findings are consistent with Figure 1, except for EZ width measures due to the limited data availability. (**a**) EZ widths at first visit from all patients are reported as data points. For patients with a non-measurable EZ, a value of 0 µm was used for plotting purposes. EZ widths from follow-up visits were also reported by simple segments, rather than best linear fit. (**b**) Inter-ocular relationship for EZ width at baseline. (**c**) CRTs at first visit from all patients are reported as data points. For patients with available follow-up visits, CRTs at all visits were fitted with linear regression for each patient and the corresponding fit is reported (light grey segments). (**d**) Inter-ocular relationship for CRT at baseline, at last visit and overall change from baseline. A similar representation is reported for the PR+RPE thickness, with changes over time in (**e**) and inter-ocular relationship in (**f**).

Baseline SD-OCT scans featured an intact EZ in 12 patients (34.3%, median age: 14.5 years, IQR: 12 to 21.8), with just a para-foveal remnant in 5 cases (see Figure 2b,c). None of the patients showed a pattern of focal loss with or without RPE changes, while the majority of eyes showed severe disruption of EZ and atrophy of the outer retina (n = 23 [65.7%], median age: 35 years, IQR: 26 to 48). Patients with intact EZ were of younger age compared to those with severe disruption (Mann–Whitney test, *p* = 0.0005), and presented a median EZ width of 564.5 µm (IQR: 485 to 903). Only five patients with preserved EZ at baseline had a follow-up examination still presenting a preserved EZ to enable measurements of change over time. The baseline and last visit were a median of 2 years apart (IQR: 1 to 2). Given the limited data availability, inter-ocular analysis was restricted to baseline and showed a moderate–strong correlation (Spearman’s rho: 0.65, 95%CI: 0.12 to 0.89, *p* = 0.025). Similarly, age-related changes were only evaluated with a cross-sectional approach and although we found a clinically meaningful estimate, the effect of age on EZ width was not statistically significant (β: −31.4 µm/year, 95%CI: −153.5 to 90.8, *p* = 0.58). Age-related changes and inter-ocular relationships of EZ width are reported in Figure 3a,b.

#### 3.4.2. Central Retinal Thickness and PR+RPE Complex

Retinal thickness data of the whole (CRT) or the outer retina (PR+RPE) were measurable in all patients with gradable OCT scans (Table 1).

After removing one patient with CMO, patients had a median CRT at baseline of 118 µm (IQR: 94 to 145) which dropped to a median of 96 µm (IQR: 83 to 129.5) at the last visit. Inter-ocular analysis (Figure 3d) showed a strong correlation for CRT in right and left eyes at baseline (Spearman’s rho: 0.91, 95%CI: 0.83 to 0.96, *p* < 0.0001), and at the last visit (Spearman’s rho: 0.80, 95%CI: 0.59 to 0.90, *p* < 0.0001). CRT changes from baseline were also strongly related (Spearman’s rho: 0.72, 95%CI: 0.45 to 0.87, *p* < 0.0001). Bland–Altman analysis (Table S2) did not show significant bias between the right and left eyes for measures at baseline, at the last visit and for change from baseline. Widths of 95% limits of agreement ranged between 76.4 µm and 91 µm. As reported in Figure 3c, age had a significant effect on CRT. Cross-sectional analysis of first visits showed a linear effect of age of β: −13.0 µm/10 years (95%CI: −22.2 to −3.9, *p* = 0.007, R^2^: 0.208). A slightly better fit was achieved by considering exponential decay, with an estimated loss of −12.6% per 10 years (95%CI: −20.7 to −4.5, *p* = 0.003, R^2^: 0.236). Similarly, longitudinal analysis showed a significant linear effect of age on CRT (β: −15.1 µm/10 years, 95%CI: −20.6 to −9.7, *p* < 0.0001, R^2^: 0.322), and a similar fit of the data with exponential decay, resulting in an estimated loss of −13.9% per 10 years (95%CI: −18.8 to −8.9, *p* < 0.0001, R^2^: 0.315).

As per CRT, PR+RPE thickness data were analysed while excluding one patient with CMO. Patients showed a median PR+RPE thickness of 41.5 µm (IQR: 25 to 70.25) at baseline, which reduced to 25 µm (IQR: 18 to 38) at the last visit. As reported in Figure 3f, a strong inter-ocular correlation of PR+RPE thickness was found at first and last visit (Spearman’s rho: 0.92, 95%CI: 0.83 to 0.96, and 0.86, 95% CI: 0.72 to 0.94, respectively, all *p* < 0.0001) and overall changes from baseline were strongly related (Spearman’s rho: 0.76, 95%CI: 0.52 to 0.87, *p* < 0.0001). As reported in Table S2, no bias was observed for PR+RPE measures, and the widths of 95% limits of agreement ranged between 27.3 µm and 35 µm. The relationship between PR+RPE and age is reported in Figure 3e. Cross-sectional data from baseline visits showed a linear effect of β: −10.6 µm/10 years (95%CI: −15.2 to −6.0, *p* = 0.001, R^2^: 0.405), and an exponential decay of −27.2% per 10 years (95%CI: −36.9 to −17.5, *p* < 0.0001, R^2^: 0.5). Similar findings were noted from the longitudinal analysis, with a significant linear effect of age (β: −12.5 µm/10 years, 95%CI: −15.8 to −9.2, *p* < 0.0001, R^2^: 0.504), and better fitting of the data with exponential decay (−33.9% per 10 years, 95%CI: −41.5 to −26.3, *p* < 0.0001, R^2^: 0.577).

### 3.5. Additional Analyses

**Figure 4:**
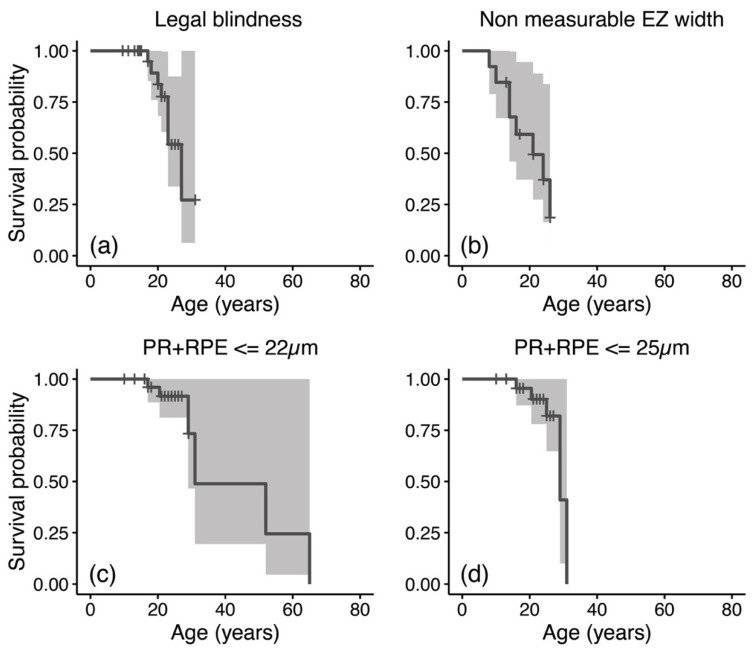
Kaplan–Meier curves for best corrected visual acuity (BCVA), ellipsoid zone (EZ) width, and photoreceptor and retinal pigment epithelium (PR+RPE) thickness. For BCVA analysis (**a**), an outcome of BCVA ≥ 1.00 LogMAR (legal blindness) was considered. For EZ width (**b**) the selected outcome was a finding of not measurable EZ. For PR+RPE thickness, a value of 22 µm was considered (**c**) and the analysis was repeated by considering the 95th percentile of PR+RPE in this sample for patients aged 30 years or more (**d**); 95% confidence intervals are also reported underneath survival curves.

Results from the survival analysis are reported in Figure 4. Findings from CRT were not included, as none of the patients in this cohort showed CRT values as low as the selected endpoint (i.e., CRT ≤ 22 µm). The median survival time for legal blindness (BCVA ≥ 1.0 LogMAR) was 27 years, whereas it was lower for EZ width (21 years). Considering an endpoint of 22 µm, PR+RPE thickness had a median survival time of 31 years. However, patients in this cohort exhibited a PR+RPE floor higher than 22 µm (see Figure 3e). This analysis was repeated while considering the 95th percentile of PR+RPE in patients 30 years and older in our cohort (i.e., 25 µm, Figure 4d), with a median survival age of 29 years.

To test any genotype–phenotype correlation, we explored differences in OCT measures and BCVAs according to variant type (i.e., missense, nonsense and frameshift). Results are reported in detail in Table S3, and we did not detect any significant differences in BCVA at baseline across variant types, and for any of the SD-OCT quantitative measures assessed.

Correlation between different SD-OCT measures and BCVA is reported in Figure S3. The significance level was set to 0.006 after Bonferroni correction (nine comparisons). CRT and PR+RPE were strongly related at the first visit (rho: 0.79, 95%CI: 0.62 to 0.89, *p* < 0.0001) and the correlation remained significant, although poorer, at the last visit (rho: 0.60, 95%CI: 0.23 to 0.79, *p* = 0.0008). Measures of EZ width at baseline (n = 12) showed stronger correlation with PR+RPE (rho: 0.69, 95%CI: 0.19 to 0.90, *p* = 0.01) than with CRT (rho: 0.47, 95%CI: −0.14 to 0.82, *p* = 0.12), but neither was statistically significant. Regarding the correlation between SD-OCT metrics and visual function, both CRT and PR+RPE had a strong negative relationship with BCVA at the first visit (−0.77, 95%CI: −0.88 to −0.59, *p* < 0.0001, and −0.87 95%CI: −0.93 to −0.76, *p* < 0.0001; respectively). Similarly, baseline EZ widths showed a strong negative correlation with BCVA (−0.76, 95%CI: −0.87 to 0.57, *p* < 0.0001).

## 4. Discussion

This study reports the largest retrospective longitudinal analysis of 47 patients with *RP2*-related X-linked RP to our knowledge, to date. These patients present with a severe phenotype, with early macular involvement and vision loss from adolescence and early adulthood. Natural history studies are required to identify potential outcome metrics for clinical trials and to provide prognostic indicators for patients. In this cohort, 24 mutations were identified, 13 of which were novel, and we found a high prevalence of missense variant c.352C>T p.(Arg118Cys) and nonsense variant c.358C>T p.(Arg120*), affecting two and five unrelated families, respectively. We did not find evidence of genotype–phenotype correlation, and this was consistent with previous literature, where considerable intra-familial variation in disease course has been reported [17,42].

Previous studies have described cross-sectional clinical findings in patients with *RP2-*retinopathy [14,16,17,42]. In an early work, Sharon and colleagues [16] reported data from 16 patients with a mean age of 27 years and BCVA of 1.0 LogMAR, which was worse than in patients with *RPGR* mutations in all age groups. In a later study, Prokish et al. assessed 81 males with X-linked RP, 26 of which had molecularly confirmed *RP2* [42]. Patients had a mean age of 30.4 years, and BCVA was more severely affected in *RP2* compared to *RPGR* cases with 8% vs. 69% displaying BCVA better than 1.0 LogMAR. Similar findings were reported by Jayasundera et al. in a study including 22 patients with *RP2* mutations, where 92% showed macular involvement, and 9 out of 11 patients had a BCVA of 0.4 LogMAR or worse by age 12 [17]. Fujinami and colleagues retrieved published data from 12 Japanese males with a mean BCVA of 1.14–1.25 LogMAR at a mean age of 31.2 years [14]. These results are in agreement with our study where the onset was by the first decade of life for the majority of patients [14,42]. By the third decade, most patients presented with a BCVA of 1.0 or worse, meeting WHO severe visual impairment and legal blindness thresholds.

Our longitudinal analysis revealed significant age-related loss of BCVA (46–49% loss per 10 years), CRT (13–14% loss per 10 years) and PR+RPE (27–34% loss per 10 years). The severe progression rate resulted in all quantitative measures showing advanced loss by the third–fourth decade of life. Longitudinal studies assessing the natural history of X-linked RP due to *RPGR* mutations, also summarised in a recent systematic review [18], reported rapid progression rates with severe visual impairment and blindness by age 40–50, with few measurable metrics, including BCVA [18]. Our longitudinal rate of BCVA loss (0.037 LogMAR/year, 95%CI: 0.032 to 0.043) was slightly faster compared to those summarised by Zada et al. [18] for *RPGR* patients of similar age at baseline (0.015 to 0.018 LogMAR/year). By comparison, a much more rapid progression rate was reported for EZ width in *RPGR* cases (173–289 µm/year) [18]; this could be explained by baseline EZ widths being much larger in the *RPGR* cohort (1936–3410 µm) compared to our *RP2* patients (565 µm), for most of whom the EZ was unmeasurable.

### Considerations for Future Clinical Trials

Genetic-based therapies may become a realistic option for X-linked RP due to mutations of *RP2* in the future [20,21,22,23], and the results of our study may inform the design of clinical trials in this area. The early onset and quick progression rates noted in *RP2* patients narrow the therapeutic intervention window to the first decade of life. Indeed, structural and functional parameters in most of our participants were dramatically affected at an early age, and this was consistent with other studies on X-linked RP showing earlier disease onset and severe progression compared to other autosomal forms of RP [17,18,43]. BCVA is usually not the most suitable outcome metric in RP due to slow progression rates and preservation of central vision until later in life [44], and more detailed functional assessments such as microperimetry are likely more accurate in capturing disease progression [45,46]. However, the dramatic progression rate in *RP2* may facilitate achievement of trial endpoints in shorter timeframes and detect actual change over test–retest variability. Yet, the early involvement of central vision may also herald issues with visual pathway development and amblyopia. This should be considered when interpreting BCVA measurements of patients with known macular involvement within the plasticity period.

Measures of EZ width are a powerful outcome metric for many inherited retinal diseases as they are objective, highly repeatable and correlate to function [18,24,47]. However, in our *RP2* study cohort, EZ-related measures provided little value to monitor progression, being measurable at baseline only in a minority of patients and quickly becoming unusable for follow-up in all patients. EZ widths would likely play a key role in monitoring progression in childhood but have little value after the first and second decades. In contrast, the thickness of the outer retina (PR+RPE) remained usable for a slightly longer period, being within its dynamic range in 50% of patients until 30–35 years of age. Our results are consistent with findings in different forms of RP and other macular disorders [32,48], suggesting that PR+RPE thickness could be a valuable quantitative and objective surrogate of central visual function. Compared to EZ width, this thickness could be extracted in a larger proportion of patients. Additionally, PR+RPE thickness was strongly related to BCVA (rho: −0.87 to −0.70) and to EZ width (rho: 0.69), considering those patients where EZ width could be measured. All quantitative measures strongly correlated between the two eyes at baseline and at the most recent visit, suggesting a high degree of inter-ocular symmetry.

The study limitations include the retrospective design, which results in a potential lack of standardisation of clinical investigations, variable imaging protocols and limited availability of tests. Nonetheless, given the rarity of the condition, this approach remains appropriate and enables the presentation of highly valuable data. Quantitative measures were extracted by one single senior grader, but all parameters considered have excellent inter- and intra-observer repeatability [18,31]. Quantitative refractive measures were available only in 10 patients (median spherical equivalent −7.7 D, IQR: −8.3 to −6.2) and were therefore deemed unavailable in this study. Similarly, visual field examinations and electrophysiology testing were available in a minority of patients and showed severe loss at baseline, and therefore were not considered in this analysis. Retinal sensitivity measures in the form of dynamic and static perimetry or microperimetry can complement retinal imaging in assessing IRDs. The use of perimetry in children with a severe phenotype has limitations, but microperimetry has high reproducibility, as well as good inter-ocular correlation, and can accurately detect disease progression in other forms of RP in younger cohorts, including *RPGR*-associated RP [49].

## 5. Conclusions

In anticipation of future trials, findings from our study provide insights into the natural history and disease progression rates in a large cohort of patients with *RP2* mutations. We found a severe disease phenotype, with early macular involvement, atrophy of the outer retina and substantial central vision loss by the third–fourth decades of life. All clinical measures assessed showed a substantial rate of loss, with EZ width being largely not measurable from 25 years of age. BCVA and outer retina thickness at the fovea seemed to offer monitoring capability for some 10 more years.

## Figures and Tables

**Table 1:** Clinical characteristics and demographics of *RP2* patients included in this cohort. Continuous variables are reported as the median and interquartile range (IQR), whereas categorical variables as count (n) and frequency (%).

	n or Median	% or IQR
**Age at first visit** (n = 47) Median (IQR), years	20	12.5–36.5
**Age at last visit** (n = 41) Median (IQR), years	27	22.08–41.5
**Age at first symptoms** (n = 14) Median (IQR), years	7	2.25–12
**Presenting symptoms** (n = 23)		
Nyctalopia, n (%)	16	69.6%
Reduced vision, n (%)	3	13.0%
Reduced vision and nyctalopia, n (%)	2	8.7%
Nystagmus, n (%)	1	4.4%
Asymptomatic, n (%)	1	4.4%
**BCVA baseline, RE** (n = 47) Median (IQR), LogMAR	0.66	0.35–1.4
**BCVA last visit, RE** (n = 41) Median (IQR), LogMAR	1.3	0.6–1.4
**Lens status baseline, RE** (n = 22)		
Phakic clear, n (%)	9	40.9%
Cataract, n (%)	8	36.4%
Pseudophakia, n (%)	5	22.7%
**Fundus photo baseline, RE** (n = 37)		
Intra-retinal pigmentation, n (%)	33	89.2%
Attenuated blood vessels, n (%)	35	94.6%
Optic disc waxy pallor, n (%)	10	27.0%
Macular changes, n (%)	22	58.5%
**FAF baseline, RE** (n = 38)		
Hyper autofluorescent ring, n (%)	9	23.8%
Central hyper autofluorescence, n (%)	6	15.8%
Severe loss of autofluorescence, n (%)	21	55.3%
**OCT metrics baseline, RE** (n = 35)		
EZ intact, n (%)	12	34.3%
Severely disrupted EZ, n (%)	23	65.7%
EZ width (n = 12)	564.5	484.8–903.3
CRT (n = 35)	119	94–145.5
PR+RPE thickness (n = 35)	43	25–71

BCVA: best corrected visual acuity; RE: right eye; FAF: fundus autofluorescence; EZ: ellipsoid zone; CRT: central retinal thickness; PR+RPE: photoreceptor and retinal pigment epithelium complex.

## Data Availability

Raw and summarised data presented in this study are reported in main and supplementary tables. Full datasets are available on request from the corresponding author.

## Supplementary Materials

The following supporting information can be downloaded at: https://www.mdpi.com/article/10.3390/jcm11236877/s1, Figure S1: Details of quantitative SD-OCT measures considered in this study (b,c) and un-segmented scan for the same patient (a). Figure S2: Position of *RP2* variants and amino-acid changes in the participating patients. Figure S3: Scatterplots of the correlation between SD-OCT quantitative measures (a–c) and structure-function relationship (d–f). Table S1: Details of *RP2* genetic variants identified in the study population (based on NM_006915.3). Table S2: Bias and Limits of agreement (LoA) at the 95% level between right and left eyes for quantitative clinical measures considered. Table S3: Results from genotype-phenotype correlation in *RP2* patients.
