# Supplementary material for: A Natural History Study of RP2-Related Retinopathy
Source: J Clin Med. 2022 Nov 22;11(23):6877. doi: 10.3390/jcm11236877 (PMC9738434; doi:10.3390/jcm11236877)
Supplement: Supplementary file 1 [file jcm-11-06877-s001.zip › jcm-1965832-supplementary.pdf]

Supplementary Tables

Table S1

Details of *RP2* genetic variants identified in the study population (based on NM_006915.3). Age at first visit and corresponding best corrected visual acuity (BCVA, LogMAR) at baseline is also reported. Novel variants are reported in bold. CF = Counting fingers; HM = hand motion; PL: perception of light; NPL: no perception of light.

#	Variant and predicted protein change	Variant type	Family ID (GC No) and – individual	BCVA at (age)
1	c.14_16delTCT (p.Phe5del)	InFrameDeletion	20016 – 1	PL (39y)
			20016 – 2	0.3 (20y)
2	**c.19A>T (p.Lys7*)**	Nonsense	17177 – 1	0.5 (11y)
			17177 – 2	0.0 (13y)
3	c.43delT (p.Ser15Argfs*31)	Frameshift	14453 – 1	0.3 (7y)
4	**c.128_140del13 (p.Ser43Metfs*3)**	Frameshift	18287 – 1	1.4 (39y)
5	**c.128delG (p.Ser43Metfs*3)**	Frameshift	28256 – 1	1.3 (26y)
6	**c.159_160insAA (p.Pro54Asnfs*5)**	Frameshift	26491 – 1	0.6 (20y)
7	**c.181C>T (p.Gln61*)**	Nonsense	26582 – 1	0.2 (7y)
8	**c.235delG (p.Ala79Leufs*11)**	Frameshift	4488 – 1	0.8 (15y)
			27172 – 1	0.7 (15y)
9	c.257G>A (p.Cys86Tyr)	Missense	26580 – 1	0.66 (13y)
10	**c.258T>A (p.Cys86*)**	Nonsense	1129 – 1	HM (62y)
11	c.338C>A (p.Ala113Asp)	Missense	20023 – 1	0.56 (12y)
12	**c.341G>A (p.Cys114Tyr)**	Missense	15222 – 1	0.3 (10y)
			15222 – 2	HM (39y)
			15222 – 3	0.4 (4y)
13	c.352C>T (p.Arg118Cys)	Missense	18241 – 1	0.8 (17y)
			20948 – 1	PL (40y)
			20948 – 2	1.4 (35y)
			20948 – 3	0.3 (16y)
			20948 – 4	PL (36y)
14	c.353G>A (p.Arg118His)	Missense	18811 – 1	PL (55y)
15	c.358C>T (p.Arg120*)	Nonsense	8 – 1	HM (59y)
			22 – 1	1 (21y)
			17759 – 1	0.6 (11y)
			18394 – 1	1.3 (22y)
			25995 – 1	0.4 (11y)
16	**c.450G>A (p.Trp150*)**	Nonsense	27392 – 1	1.3 (29y)
17	**c.460G>T (p.Glu154*)**	Nonsense	34 – 1	0.6 (18y)
			34 – 2	0.5 (11y)
			34 – 3	HM (43y)
			18099	0.24 (13y)
18	**c.568_569delinsG (p.Pro190Glufs*48)**	Frameshift	49 – 1	HM (46y)
			49 – 2	0.3 (24y)
19	**c.685_691del7 (p.Gln229Alafs*7)**	Frameshift	5284 – 1	CF (49y)
			5284 – 2	0.5 (14y)
			5284 – 3	1.4 (37y)
20	c.798_801del4 (p.Thr267Argfs*5)	Frameshift	5072 – 1	1.4 (44y)
21	**c.969+3A>T**	SpliceSite	16814 – 1	0 (10y)
			16814 – 2	0.2 (21y)
22	Deletion of exon 5	Deletion	4300 – 1	1.00 (19y)
			25452 – 1	0.3 (26y)
23	Deletion of exons 1-5	Deletion	21172 – 1	0.3 (6y)
			21172 – 2	0.62 (8y)
24	Deletion of *RP*2	Deletion	18829 – 1	1.4 (20y)
			26353 – 1	0.5 (19y)

Table S2

Bias and Limits of agreement (LoA) at the 95% level between right and left eyes for quantitative clinical measures considered. Ellipsoid Zone width data are not reported as quantitative measures were only available in a minority of the cohort. BCVA = Best corrected visual acuity; CRT = Central retinal thickness; PR+RPE = Photoreceptor and retinal pigment epithelium complex; 95%CI = 95% confidence interval.

Clinical Measures		Baseline	Last visit	Raw change from baseline
BCVA (LogMAR)	Bias (95%CI)	-0.02 (-0.09 to 0.05)	-0.04 (-0.14 to 0.06)	-0.05 (-0.16 to 0.07)
	95% LoA	-0.42 to 0.38	-0.55 to 0.46	-0.60 to 0.51
CRT (μm)	Bias (95%CI)	-3.2 (-10.9 to 4.4)	-1.9 (-11.3 to 7.5)	-0.2 (-8.2 to 7.9)
	95% LoA	-44.7 to 38.3	-47.4 to 43.6	-38.4 to 38.0
PR+RPE (μm)	Bias (95%CI)	1.8 (-1.4 to 5.0)	0.1 (-3.4 to 3.6)	-1.8 (-4.7 to 1.0)
	95% LoA	-15.7 to 19.3	-16.9 to 17.2	-15.5 to 11.8

Table S3

Results from genotype-phenotype correlation in *RP2* patients. Differences in best corrected visual acuity (BCVA) and quantitative SD-OCT measures were evaluated according to variant type. Unless specified otherwise, median and (interquartile range) are reported for continuous variables. Differences were tested with non-parametric ANOVA (Kruskal-Wallis test).

	**Whole cohort**	**Missense variants**	**Nonsense variants**	**Frameshift variants**	**P-value**
**N** (%)	47 (100)	11 (23.4)	14 (29.8)	12 (25.5)	-
**Age at first visit** (y)	20 (12.5-36.5)	17 (12.5-37.5)	15.5 (11-27.25)	25 (15-40.25)	0.58
**BCVA baseline, RE**					
BCVA, LogMAR (n=47)	0.56 (0.3-1)	0.8 (0.48-2.75)	0.6 (0.43-1.3)	1.05 (0.58-1.4)	0.19
**SD-OCT metrics baseline, RE**					
CRT (n=35)	119 (94-145.5)	140 (98-147)	121 (88-153)	108 (92.75-117.8)	0.76
PR+RPE (n=35)	43 (25-71)	57 (36-69.5)	40 (27.5-73)	30.5 (26.25-51.25)	0.79

Supplementary Figures

Figure S1

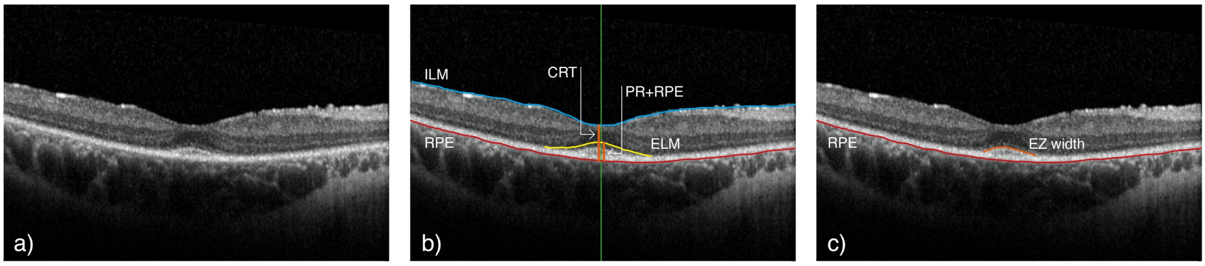

Details of quantitative SD-OCT measures considered in this study (b and c) and un-segmented scan for the same patient (a).

After identification of the trans-foveal B-scan retinal thickness was measured at the fovea along the green line in (b) as vertical distance between: inner limiting membrane (ILM) and retinal pigment epithelium (RPE) for central retinal thickness (CRT); and between external limiting membrane (ELM) and RPE for photoreceptor and RPE complex (PR+RPE). EZ width is shown in (c), the layer was followed temporal and nasally until no more distinguishable from RPE.

Figure S2

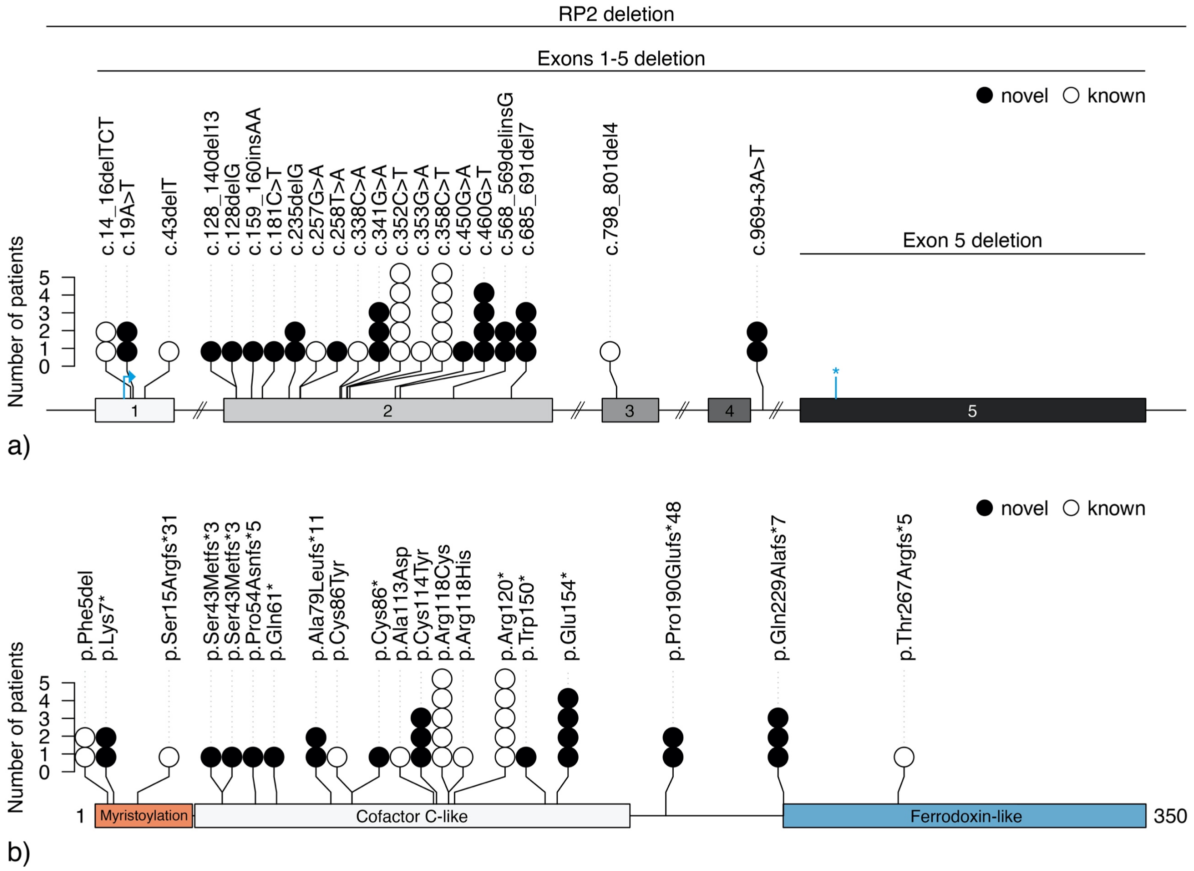

Position of RP2 variants and amino-acid changes in the participating patients.

(a) reports a schematic diagram of RP2 gene (exon 5 not in scale for representation purpose). Mutations are reported as lollipops at the corresponding position. (b) schematic diagram of RP2 protein structure, with 3 domains (Jayasundera et al., 2010 [17]). Novel variants are reported as full-black lollipop.

Figure S3

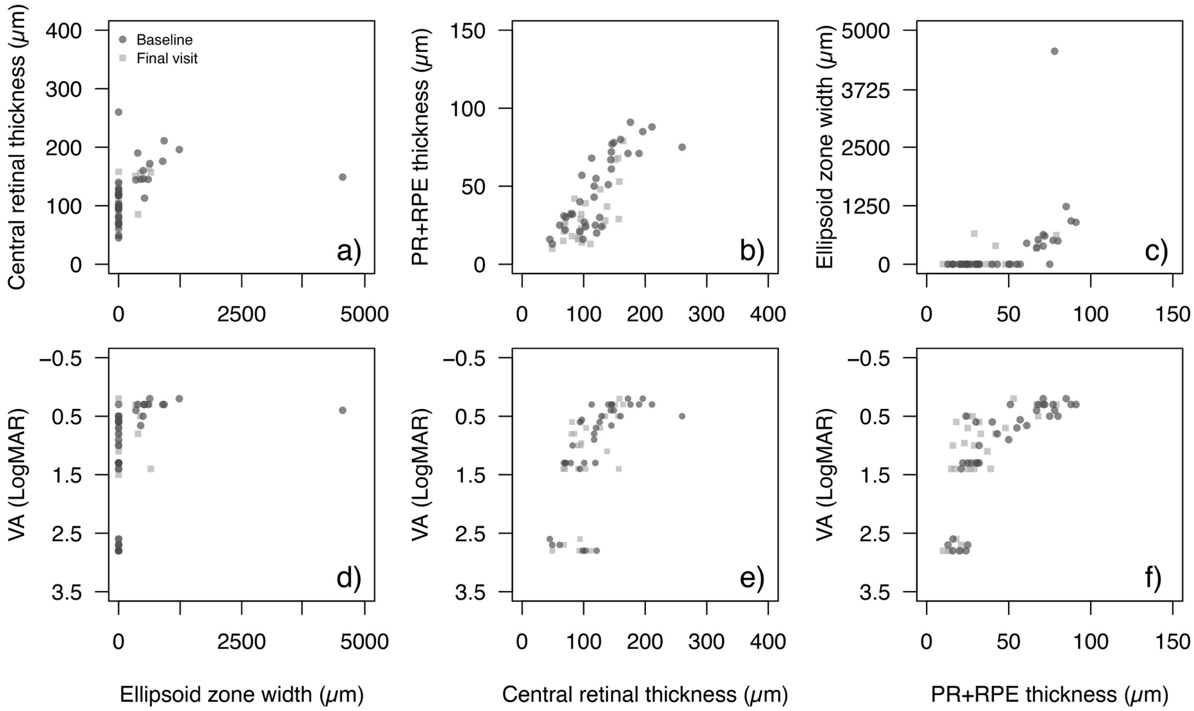

Scatterplots of the correlation between SD-OCT quantitative measures (a, b, and c) and structure-function relationship (d, e, and f).

Data are reported at the first and last visit and colour-coded accordingly. Only for plotting purposes, a finding of non-measurable EZ was plotted by passing a value equal to 0µm, yet correlation coefficients were computed only with real measures (see text).
